# Can multiple SNP testing in *BRCA2* and *BRCA1* female carriers be used to improve risk prediction models in conjunction with clinical assessment?

**DOI:** 10.1186/1472-6947-14-87

**Published:** 2014-10-01

**Authors:** Mattia CF Prosperi, Sarah L Ingham, Anthony Howell, Fiona Lalloo, Iain E Buchan, Dafydd Gareth Evans

**Affiliations:** Institute of Population Health, Centre for Health Informatics, University of Manchester, Manchester, UK; Genesis Prevention Centre, University Hospital of South Manchester, Manchester, UK; Department of Genetic Medicine, Manchester Academic Health Science Centre, St. Mary’s Hospital, University of Manchester, Manchester, UK

**Keywords:** Breast cancer, *BRCA1*, *BRCA2*, Single nucleotide polymorphism, Cox regression, Random survival forests, Survival analysis, Prognostic model, Concordance index

## Abstract

**Background:**

Several single nucleotide polymorphisms (SNPs) at different loci have been associated with breast cancer susceptibility, accounting for around 10% of the familial component. Recent studies have found direct associations between specific SNPs and breast cancer in *BRCA1/2* mutation carriers. Our aim was to determine whether validated susceptibility SNP scores improve the predictive ability of risk models in comparison/conjunction to other clinical/demographic information.

**Methods:**

Female *BRCA1/2* carriers were identified from the Manchester genetic database, and included in the study regardless of breast cancer status or age. DNA was extracted from blood samples provided by these women and used for gene and SNP profiling. Estimates of survival were examined with Kaplan-Meier curves. Multivariable Cox proportional hazards models were fit in the separate *BRCA* datasets and in menopausal stages screening different combinations of clinical/demographic/genetic variables. Nonlinear random survival forests were also fit to identify relevant interactions. Models were compared using Harrell’s concordance index (1 - c-index).

**Results:**

548 female *BRCA1* mutation carriers and 523 *BRCA2* carriers were identified from the database. Median Kaplan-Meier estimate of survival was 46.0 years (44.9-48.1) for *BRCA1* carriers and 48.9 (47.3-50.4) for *BRCA2*. By fitting Cox models and random survival forests, including both a genetic SNP score and clinical/demographic variables, average 1 - c-index values were 0.221 (st.dev. 0.019) for *BRCA1* carriers and 0.215 (st.dev. 0.018) for *BRCA2* carriers.

**Conclusions:**

Random survival forests did not yield higher performance compared to Cox proportional hazards. We found improvement in prediction performance when coupling the genetic SNP score with clinical/demographic markers, which warrants further investigation.

## Background

*BRCA1* and *BRCA2* are major susceptibility genes that confer high lifetime risks for both breast and ovarian cancer. Deleterious mutations in these autosomal dominant cancer genes account for approximately 15-20% of the familial component of breast cancer [1–3]. The variable penetrance exhibited by these *BRCA* mutations suggest other genetic factors to be present [4], and several studies have now identified a large number of breast cancer susceptibility alleles [5–7]. Genome association studies had identified until recently 19 common variants at 18 loci that are associated with breast cancer susceptibility [5, 7] though the risk attributed to each of these single nucleotide polymorphisms (SNPs) are often modest and largely remain unexplained [6]. More recent studies into these polymorphisms have found direct associations between specific SNPs and breast cancer in *BRCA1/2* mutation carriers; *TOX3, FGFR2, MAP3K, LSP1*, 2q35, *SLC4A7*, 1p11.2, 5p12, 6q25.1 loci have all been associated with increased risk in breast cancer for *BRCA2* mutation carriers [6, 7]. Antoniou *et al.* [6] further determined *TOX3*, 2q35, and 6q25.1 were polymorphisms that increased risk for *BRCA1* mutation carriers. However, a recent study by Ingham *et al.* [8] found the 18 validated breast cancer susceptibility SNPs do not differentiate the risks of breast cancer in those with *BRCA1* mutations.

Some genetic modifiers may in themselves influence breast cancer risk factors rather than be directly associated; such as the genetic component associated with high mammographic density [4, 9]. A recent study by Mitchell *et al.* looking at mammographic density in 206 *BRCA1* and *BRCA2* carriers compared to non-carriers found a significant association between increased breast cancer risk and increasing density in *BRCA1/2* carriers [9].

Alongside risk factors with a genetic component there are several hormonal risk factors that are thought to be associated with breast cancer both among the general population and those with hereditary breast cancer [10]. Correlations have been made between changes in breast mitotic/apoptotic activity and alterations in hormone levels across the menstrual cycle, and that if the levels of oestrogen and progesterone are reduced then the risk of breast cancer is reduced [11, 12]. Though some debate surrounds the association of these factors with breast cancer among *BRCA1/2* carriers, with studies finding an association only in *BRCA1* mutation carriers [13] and other finding no association [12]. Modifiable factors, such as body mass index (BMI) are also thought to influence the risk of breast cancer. Obesity has a well-documented association with breast cancer in the general population, due to influence of biological pathways [14], and postmenopausal weight gain has been associated with increased risk among *BRCA* carriers [15].

At present, several personalised risk prediction models have been developed using familial, demographic, clinical, laboratory, genetic information domains, with a few combinations thereof [8, 16–19], as for instance the Gail, BOADICEA or IBIS methods [20], as well as more specific studies as surveys on gene expression markers [21], and use of machine learning for predicting recurrence or re-defying subtypes [22, 23].

The aim of this study was to determine whether validated susceptibility SNPs improve the predictive ability of risk models in conjunction and comparison to demographic and clinical information.

## Methods

### Study population

Patients included in this study were *BRCA1* and *BRCA2* female pathogenic mutation carriers ascertained from the Genetic Medicine department, St Mary’s Hospital, Manchester, UK. This clinic is one of the largest specialist genetics departments within the UK, and all families with a history of breast or ovarian cancer within the North West region are referred. Patients were included in this study regardless of breast cancer status or age. Dates of birth were taken from the information collected at time of family referral to the genetics department. Cases of breast cancer were confirmed by means of hospital records or the North West Cancer Intelligence Service. Dates of last follow-up were either date of breast cancer diagnosis or date the woman was last in contact with the genetics department or other NHS service or date of death.

### Ethics statement

This research has been performed in accordance with the Declaration of Helsinki. The NHS Health Research Authority, National Health Research Ethics Committee North West, Greater Manchester Central (Barlow House, 4 Minshull Street, Manchester, M1 3DZ), reviewed this study and gave ethical approval; the Research Ethics Committee reference number is 10/H1008/24, dated 11^th^ July 2013. Written informed consent was obtained from all study participants (none minor at the time of enrolment).

### DNA testing

DNA was extracted from blood samples provided by women attending the genetic clinics, using DNA Sanger sequencing and multiplex ligation-dependent probe amplification analysis for gene and SNP profiling; *BRCA1* and *BRCA2* mutations were identified as well as the presence of any of the 18 tested breast cancer SNPs. Overall breast cancer SNP risk scores were calculated for each woman using the methods as recorded in the article Ingham *et al.* [8].

### Statistical models

The study population was stratified by *BRCA* type (1 or 2) and menopausal stage (ovulating vs. menopause). Incidence of breast cancer was calculated for the strata, as well as Kaplan-Meier [24] estimates of survival. Main-effect multivariable Cox proportional hazards (CPH) [25] models were fit in the separate *BRCA* data sets and then in the menopausal stages. End-point was the time to cancer, censored by the current age (or loss to follow up, or death for other causes). Proportional hazards assumption was tested *via* weighted residuals [26]. Variables included in the analyses were (see Table 1): year of birth, Manchester score [27] (transformed using the inverse hyperbolic sine), BMI; parity; age of menarche; age of menopause; age of first full-term pregnancy; oral contraception usage; time of diagnosis of an ovarian cancer followed up by oophorectomy (if any); time of mastectomy (if any); SNPs rs614367, rs704010, rs713588, rs889312, rs909116, rs1011970, rs1156287, rs1562430, rs2981579, rs3757318, rs3803662, rs4973768, rs8009944, rs9790879, rs10995190, rs11249433, rs13387042, rs10931936, genetic predisposition score (GPS), calculated on the mentioned SNPs according to Ingham *et al.* [8] Missing values were preliminarily analysed by means of univariable CPH, comparing Akaike information criterion (AIC) [28] and coefficient p-values of models with median/modes imputation vs. stratification into quartiles and addition of a category for those values which were missing. The following CPH models were fit for each population stratum: (i) GPS; (ii) GPS + year of birth + Manchester score + BMI + parity + age of menarche + age of menopause + age of full-term pregnancy + oral contraception usage + oophorectomy + mastectomy; (iii) SNPs; (iv) SNPs + year of birth + Manchester score + BMI + parity + age of menarche + age of menopause + age of full-term pregnancy + oral contraception usage + oophorectomy + mastectomy; (v) year of birth + Manchester score + BMI + parity + age of menarche + age of menopause + age of full-term pregnancy + oral contraception usage + oophorectomy + mastectomy; (vi) all variables. CPH models (ii), (iii), (iv) and (vi) were feature-selected using a forward/backward stepwise heuristic driven by AIC [29]. Nonlinear random survival forests (RSF) [30] were also fit on all variables to identify putative variable interactions (333 trees, choosing the log-rank splitting rule). Table 1 summarises which variables were used for each model. CPH and RSF were compared using the complementary value of Harrell’s concordance index (1 - c-index) [31] and the area under the receiver operating characteristic (AUROC) [32], under a bootstrap-based (100 resampled sets, using the out-of-bag predictions) method of extra-sample error estimation [33].

**Table 1:** **List of variables used in the study (for both BRCA1 and BRCA2 populations), data types, and variable inclusion in Cox proportional hazards models (i) to (vi)**

***Variable***	***Data type***	***Model (i)***	***Model (ii)***	***Model (iii)***	***Model (iv)***	***Model (v)***	***Model (vi)***
Genetic predisposition score (GPS)	Numeric	✓	✓				✓
Year of birth	Numeric		✓		✓	✓	✓
Manchester score	Numeric (inverse hyperbolic sine scale)		✓		✓	✓	✓
Body mass index (BMI)	Numeric		✓		✓	✓	✓
Parity	Quartiles (q1 … q4) + missing category		✓		✓	✓	✓
Age of menarche	Quartiles (q1 … q4) + missing category		✓		✓	✓	✓
Age of menopause	Quartiles (q1 … q4) + missing category + ovulating stratum		✓		✓	✓	✓
Age of first full-term pregnancy	Quartiles (q1 … q4) + missing category + never had full term pregnancy		✓		✓	✓	✓
Oral contraception usage	Quartiles (q1 … q4)		✓		✓	✓	✓
Oophorectomy	Binary (yes vs. no)		✓		✓	✓	✓
Mastectomy	Binary (yes vs. no)		✓		✓	✓	✓
Individual single nucleotide polymorphisms (SNPs, rs614367, rs704010, rs713588, rs889312, rs909116, rs1011970, rs1156287, rs1562430, rs2981579, rs3757318, rs3803662, rs4973768, rs8009944, rs9790879, rs10995190, rs11249433, rs13387042, rs10931936)	Binary (yes vs. no)			✓	✓		✓

All analyses were carried out using the R software [34].

## Results

The *BRCA1* population included 548 subjects, whilst the *BRCA2* population 523. Table 2 shows population characteristics stratified by *BRCA* type and menopausal stage.

**Table 2:** **Characteristics of the study population**

Variable	Median (interquartile range) or N (%)					
	BRCA1			BRCA2		
	Menopause (n = 200)	Ovulating (n = 113)	All (n = 548)*	Menopause (n = 195)	Ovulating (n = 93)	All (n = 523)*
Year of birth	1955 (1946-1962)	1963 (1952-1979)	1959 (1948-1969)	1952 (1944-1959)	1963 (1952-1971)	1957 (1947-1966)
Manchester score	32 (22.5-40)	28 (20-38)	29 (20-40.75)	26 (20-35)	27 (20-36)	26 (20-35)
BMI	24.07 (22.34-28.18)	22.52 (21.39-26.71)	23.7 (21.6475-27.3125)	25.12 (22.52-28.565)	24.225 (21.975-27.02)	24.84 (22.355-28.305)
Parity	2 (2-3)	1 (0-2)	2 (1-3)	2 (0-9)	2 (0-9)	2 (0-9)
Age of menarche	13 (12-14.25)	13 (12-14)	13 (12-14)	13 (12-14)	13 (12-14)	13 (12-14)
Age of menopause	43 (38-46)	44 (40-48)**	43 (39-47)**	45 (40.5-48.5)	45 (39.5-49.5)**	45 (40-49)**
Age of full-term pregnancy	24 (21-28)	23 (20-26.25)	24 (21-28)	24 (21-28)	23 (20.25-27)	24 (21-28)
Oral contraception usage	5 (1-10)	7 (2.75-11)	5 (2-10)	5 (1-10)	5 (1-10)	5 (1-10)
Genetic predisposition score	0.98 (0.70-1.29)	0.95 (0.63-1.17)	0.95 (0.67-1.27)	0.83 (0.63-1.17)	1.17 (0.77-1.62)	0.90 (0.68- 1.33)
Oophorectomy	70 (35%)	0 (0%)	71 (12.96%)	88 (45.13%)	0 (0%)	102 (19.50%)
Mastectomy	35 (17.5%)	7 (6.19%)	49 (8.94%)	17 (9.74%)	0 (0%)	17 (3.25%)
Time of observation (years)	48.04 (42.74-53.76)	30.83 (28.0-38.90)	41.26 (35.83-49.38)	50.68 (44.77-57.70)	33.66 (29.13-42.49)	44.37 (38.02-51.78)
No. of events	92 (46.00%)	88 (77.88%)	321 (58.58%)	105 (53.85%)	72 (77.42%)	323 (61.76%)

*includes also women with unknown menopausal stage status.

**women may have had menopause after a breast cancer diagnosis.

Incidence of breast cancer for all *BRCA1* carriers was 321 events per 23,649 person-years of follow-up (PYFY), i.e. 0.014 (95% confidence interval, CI 0.012 0.015). It was 92/9,872 (0.009, 95% CI 0.008-0.011) and 88/3,770 (0.023, 95% CI 0.019-0.029) for menopause and ovulating strata, respectively. The median (95% CI) Kaplan-Meier estimate of survival time to breast cancer was 46.0 (44.9-48.1) years in the whole *BRCA1* population, 53.7 (52.0-60.7) for menopause stratum, and 35.5 (32.9-38.3) for the ovulating population (p > 0.0001, log-rank test). Women diagnosed with an ovarian cancer who underwent an oophorectomy had a higher survival probability than those who did not (p > 0.0001, log-rank test). At age 50 years, probability (95% CI) of survival was 0.82 (0.70-0.96) for those who had oophorectomy (71 women, 12 breast cancer events), *versus* 0.34 (0.30-0.39) for the others. At age 60 it was 0.59 (0.40-0.86) *versus* 0.19 (0.15-0.24). There was one case of breast cancer after risk reducing mastectomy (out of 49 women operated).

**Figure 1:**
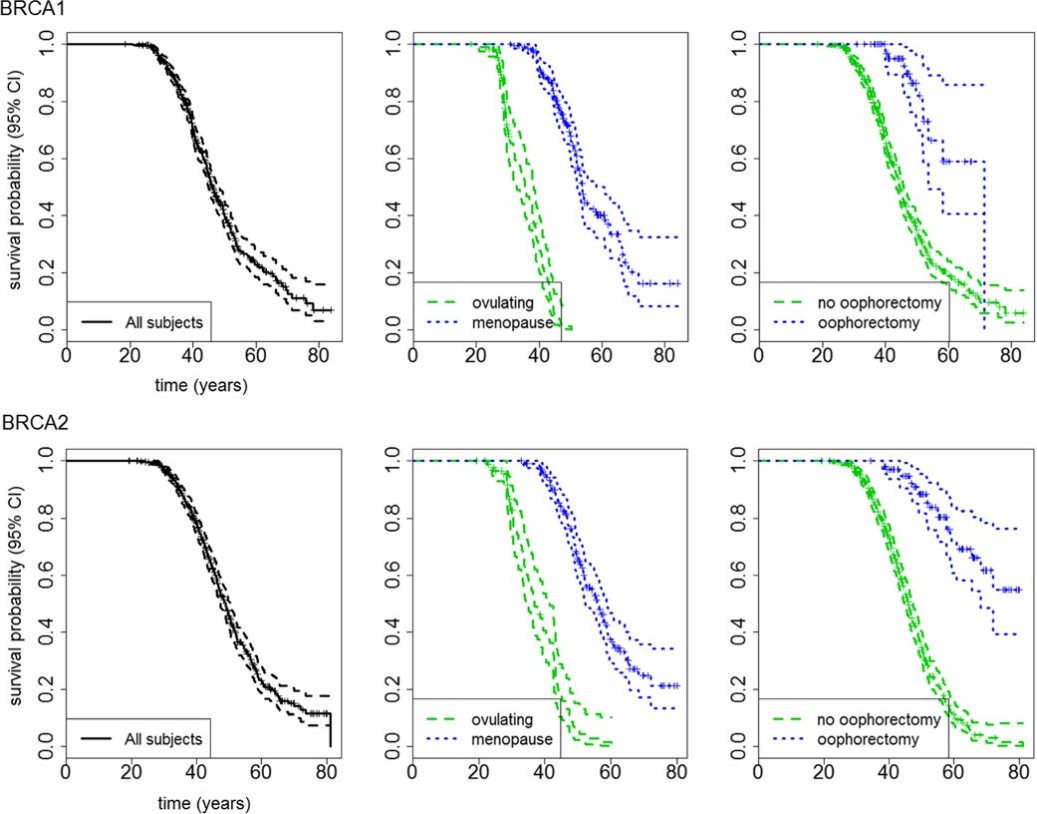
**Kaplan-Meier estimates of being cancer-free for BRCA1 (upper panels) and BRCA2 (lower panels) carriers: overall, stratified by menopausal stage, and by oophorectomy.**

Incidence of cancer for all *BRCA2* carriers was 323 events per 23,796 person-years of follow-up (PYFY), i.e. 0.014 (95% confidence interval, CI 0.012 0.015). It was 105/10,120 (0.010, 95% CI 0.008-0.012) and 72/3,265 (0.022, 95% CI 0.017- 0.028) for menopause and ovulating strata, respectively. The median (95% CI) Kaplan-Meier estimate of survival time was 48.9 (47.3-50.4) years in the whole *BRCA2* population, 56.3 (52.3-58.7) for menopause stratum, and 36.8 (34.9-41.4) for the ovulating population (p > 0.0001, log-rank test). Women who underwent an oophorectomy had a higher survival probability than those who did not (p > 0.0001, log-rank test). At age 50 years, probability (95% CI) of survival was 0.88 (0.82-0.95) for those who had oophorectomy (102 women, 23 breast cancer events), *versus* 0.34 (0.30-0.40) for the others. At age 60 it was 0.70 (0.59-0.83) *versus* 0.11 (0.07-0.15). As in the *BRCA1* population, there was only one case of breast cancer after risk reducing mastectomy (out of 17 women operated). Figure 1 shows Kaplan-Meier graphs for the whole *BRCA1/2* population, for the menopausal stage strata, and for those who had/had not oophorectomy after the diagnosis of an ovarian cancer.

**Figure 2:**
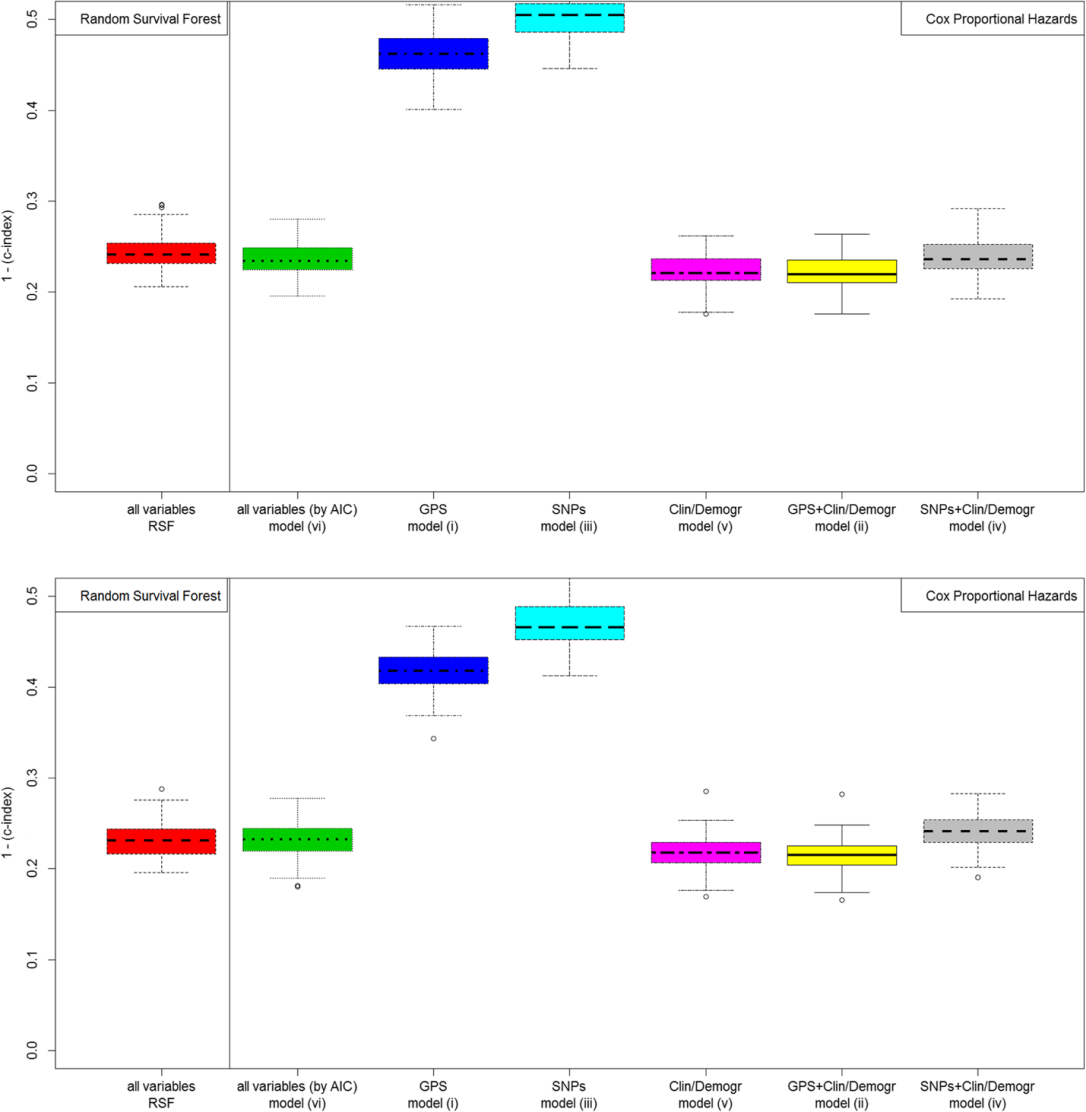
**Model selection results for** ***BRCA1*** **(upper panel) and** ***BRCA2*** **(lower panel) data sets, comparing c-index performance of Cox regression models (i) through (vi) and random survival forest (RSF).** Boxplots drawn upon out-of-bag predictions (100 resampled sets).

**Figure 3:**
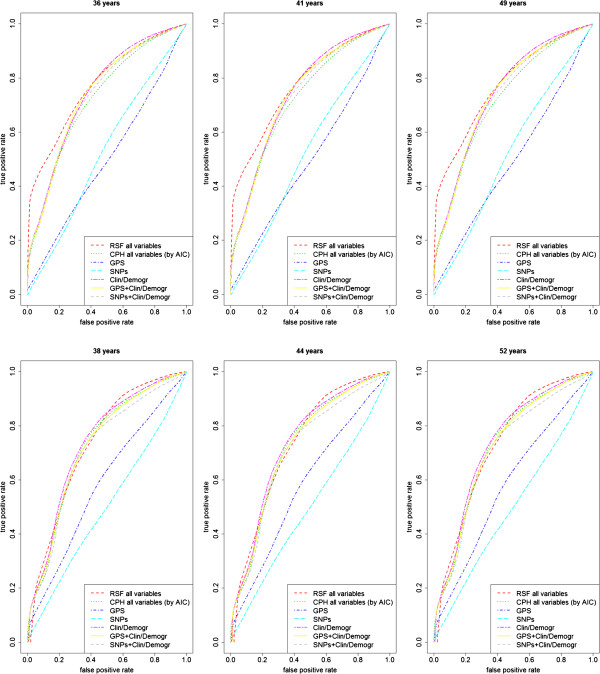
**Model selection results for** ***BRCA1*** **(upper panel) and** ***BRCA2*** **(lower panel) data sets, comparing AUROC performance of Cox regression models (i) through (vi) and random survival forest (RSF).** Time points correspond to the quartiles of the overall population observation time distribution. Curves drawn upon out-of-bag predictions (15 resampled sets).

When applying models (i) through (vi) and RSF on the whole *BRCA1* population, using the out-of-bag estimator, average (st. dev.) 1 - c-index values of models were (see Table 3), respectively, 0.468 (0.037), 0.221 (0.019), 0.504 (0.026), 0.238 (0.019), 0.222 (0.019), 0.236 (0.018), 0.243 (0.019). When applying models (i) through (vi) and RSF on the whole *BRCA2* population, using the out-of-bag estimator, average (st. dev.) 1 - c-index values of models were, respectively, 0.417 (0.021), 0.215 (0.018), 0.469 (0.028), 0.241 (0.019), 0.217 (0.018), 0.232 (0.019), 0.230 (0.019). The best model was therefore (ii), including GPS and clinical/demographic variables. The hypothesis of a lower difference in mean with respect to model (ii) for all other models could be rejected, except for model (i) and (iii), which included only genetic variables (all p > 0.0001 for both *BRCA1* and *BRCA2*, Student’s t-test corrected for sample overlap from multiple validation). Notably a re-calibrated SNP score, i.e. models (iii) and (iv), did not perform as well as the GPS. Consistent results were obtained by looking at the AUROC in the 1^st^, 2^nd^ and 3^rd^ quartiles of observation times. The AUROC estimation was performed on a smaller out-of-bag sample (333 out-of-bag instances) for computational reasons. Figures 2 and 3 show c-index/AUROC graphs for *BRCA1/2* sets based on the out-of-bag estimator. Similar figures were obtained when stratifying for the menopausal stage (data not shown).

**Table 3:** **Average (st. dev.) 1 - c-index performance results of cox proportional hazards and random survival forest models as estimated by collating out-of-bag distributions from 100 bootstrap runs**

***Model***	***Average (st.dev.) 1 - c-index***	
	***BRCA1***	***BRCA2***
(i) GPS	0.468 (0.037)*	0.417 (0.021)*
(ii) GPS + Clin./Demogr.	**0.221 (0.019)**	**0.215 (0.018)**
(iii) SNPs	0.504 (0.026)*	0.469 (0.028)*
(iv) SNPs + Clin./Demogr.	0.238 (0.019)	0.241 (0.019)
(v) Clin./Demogr.	0.222 (0.019)	0.217 (0.018)
(vi) all variables (by AIC)	0.236 (0.018)	0.232 (0.019)
Random survival forest (all variables)	0.243 (0.019)	0.230 (0.019)

*p > 0.0001 as compared to model (ii) by an adjusted t-test. Values in bold show the best performance.

Tables 4 and 5 report relative hazards obtained by fitting Cox model (ii) on *BRCA1* and *BRCA2* populations, overall and stratified by menopausal stage. There was a calendar year of birth effect, increasing the risk of cancer for both *BRCA1/2* carrier cohorts (RH ranging from 1.06 to 1.08, p > 0.0005 across all strata). The Manchester score had a protective effect in the *BRCA1* menopause stratum (RH = 0.35, p = 0.0006) and showed the same trend in the whole *BRCA1* population (RH = 0.8, p = 0.1), but the RH directions were not consistent across all strata as well as significance levels. The GPS score had a protective effect in the whole *BRCA1* population and in the ovulating strata (RH 0.76/0.58, p > 0.015), and was associated to a higher hazard of breast cancer in the *BRCA2* whole population (RH = 1.33, p = 0.035).

**Table 4:** **Multivariable cox regression fit on** ***BRCA1*** **data set, overall and stratified by menopausal stage, with covariate set based on model (ii)**

Variable	BRCA1					
	All subjects		Menopause		Ovulating	
	AIC = 3175		AIC = 765		AIC = 591	
	PH p-value > 0.0001		PH p-value = 0.0111		PH p-value = 0.5040	
	RH (95% CI)	p-value	RH (95% CI)	p-value	RH (95% CI)	p-value
Year of birth	1.06 (1.05-1.08)	>0.0001	1.06 (1.03-1.09)	>0.0001	1.08 (1.04-1.11)	>0.0001
Manchester score	0.8 (0.61-1.05)	0.1013	0.35 (0.19-0.64)	0.0006	1.56 (0.83-2.91)	0.1635
GPS	0.76 (0.61-0.95)	0.0151	0.88 (0.54-1.45)	0.6248	0.58 (0.37-0.9)	0.0148
BMI	1.02 (0.98-1.06)	0.3942	1.01 (0.94-1.07)	0.844	1.13 (1.02-1.25)	0.0229
Parity q2 vs. q1	1.32 (0.91-1.89)	0.1389	1.99 (0.91-4.37)	0.0846	0.72 (0.32-1.63)	0.4298
Parity q3 vs. q1	1.21 (0.8-1.82)	0.377	1.24 (0.52-2.96)	0.6246	0.85 (0.36-2.04)	0.7228
Parity q4 vs. q1	1.61 (1.01-2.56)	0.046	1.88 (0.65-5.44)	0.2434	1.21 (0.43-3.4)	0.724
Parity missing vs. q1	1.22 (0.43-3.52)	0.7066	N/A	N/A	3.16 (0.34-28.9)	0.3089
Age of menarche q2 vs. q1	1.17 (0.82-1.67)	0.3914	1.14 (0.58-2.26)	0.6979	0.43 (0.17-1.07)	0.0695
Age of menarche q3 vs. q1	1.02 (0.67-1.54)	0.9345	1.02 (0.45-2.32)	0.9638	0.87 (0.45-1.71)	0.6918
Age of menarche q4 vs. q1	0.82 (0.55-1.23)	0.3437	1.34 (0.71-2.54)	0.3682	0.94 (0.4-2.16)	0.8772
Age of menarche missing vs. q1	1.12 (0.71-1.74)	0.6307	2.63 (0.86-8.03)	0.0902	1.49 (0.58-3.81)	0.4094
Age of menopause q2 vs. q1	0.37 (0.19-0.72)	0.0036	0.19 (0.09-0.43)	>0.0001		
Age of menopause q3 vs. q1	0.39 (0.22-0.7)	0.0015	0.21 (0.1-0.45)	>0.0001		
Age of menopause q4 vs. q1	0.33 (0.18-0.6)	0.0003	0.13 (0.06-0.28)	>0.0001		
Age of menopause missing vs. q1	0.64 (0.39-1.04)	0.0708				
Age of menopause not yet* vs. q1	4.48 (2.74-7.33)	>0.0001				
Age of full-term pregnancy q2 vs. q1	1.22 (0.87-1.71)	0.2594	1.96 (0.98-3.91)	0.0576	1.06 (0.45-2.49)	0.8908
Age of full-term pregnancy q3 vs. q1	1.37 (0.96-1.96)	0.079	2.01 (0.94-4.32)	0.0734	2.06 (0.97-4.38)	0.0615
Age of full-term pregnancy q4 vs. q1	1.22 (0.81-1.84)	0.3512	2.8 (1.16-6.76)	0.0221	0.95 (0.34-2.64)	0.9163
Age of full-term pregnancy missing vs. q1	1.11 (0.7-1.79)	0.652	1.31 (0.45-3.8)	0.6245	0.98 (0.34-2.83)	0.9744
Age of full-term pregnancy not yet** vs. q1	2.17 (0.98-4.79)	0.0552	N/A	N/A	2.69 (0.77-9.34)	0.1198
Oral contraception usage q2 vs. q1	0.64 (0.39-1.04)	0.0704	0.4 (0.18-0.9)	0.0263	1.14 (0.39-3.33)	0.8105
Oral contraception usage q3 vs. q1	1.07 (0.71-1.62)	0.7575	0.48 (0.23-1)	0.0495	4.77 (1.77-12.83)	0.002
Oral contraception usage q4 vs. q1	1 (0.65-1.54)	0.9936	0.98 (0.46-2.07)	0.9571	2.13 (0.82-5.54)	0.1211
Oral contraception usage missing vs. q1	1.1 (0.72-1.66)	0.6686	0.4 (0.19-0.87)	0.0207	3.94 (1.42-10.94)	0.0084
Mastectomy	0.04 (0.01-0.31)	0.0018	0.05 (0.01-0.39)	0.0044	N/A	N/A
Oophorectomy	0.31 (0.16-0.58)	0.0003	0.3 (0.15-0.62)	0.0012	N/A	N/A

N/A: could not be fit in the model; q1 … q4: first to fourth age quartile, with the first being the youngest (~40 years old); *Ovulating; **Never had full term pregnancy.

**Table 5:** **Multivariable cox regression fit on BRCA2 data set, overall and stratified by menopausal stage, with covariate set based on model (ii)**

Variable	BRCA2					
	All subjects		Menopause		Ovulating	
	AIC = 3137		AIC = 881		AIC = 475	
	PH p-value = 0.03497		PH p-value = 0.00125		PH p-value = 0.11892	
	RH (95% CI)	p-value	RH (95% CI)	p-value	RH (95% CI)	p-value
Year of birth	1.07 (1.05-1.08)	>0.0001	1.07 (1.04-1.11)	>0.0001	1.06 (1.02-1.09)	0.0005
Manchester score	1.04 (0.76-1.43)	0.8048	1 (0.61-1.65)	0.9862	1.49 (0.56-3.98)	0.4228
GPS	1.33 (1.1-1.61)	0.0038	1.33 (0.93-1.91)	0.119	0.88 (0.51-1.51)	0.6416
BMI	0.99 (0.94-1.03)	0.5491	0.99 (0.92-1.06)	0.6694	1.03 (0.92-1.16)	0.5714
Parity q2 vs. q1	0.9 (0.66-1.24)	0.5307	1.4 (0.73-2.67)	0.3096	1.06 (0.38-2.99)	0.9083
Parity q3 vs. q1	0.89 (0.6-1.34)	0.5817	1.01 (0.46-2.21)	0.9808	1.51 (0.36-6.31)	0.5705
Parity q4 vs. q1	0.78 (0.47-1.28)	0.3182	0.9 (0.38-2.13)	0.8112	1.32 (0.34-5.12)	0.6914
Parity missing vs. q1	0.15 (0.06-0.38)	>0.0001	N/A	N/A	N/A	N/A
Age of menarche q2 vs. q1	1.36 (0.94-1.97)	0.1007	2.53 (1.47-4.35)	0.0008	1.57 (0.64-3.84)	0.3251
Age of menarche q3 vs. q1	1.39 (0.94-2.07)	0.0984	1.76 (0.95-3.27)	0.0742	2.43 (0.92-6.45)	0.074
Age of menarche q4 vs. q1	1.25 (0.83-1.88)	0.2822	1.51 (0.83-2.76)	0.1803	2.03 (0.79-5.25)	0.1422
Age of menarche missing vs. q1	2.32 (1.43-3.78)	0.0007	1.54 (0.18-13.15)	0.6947	1.06 (0.24-4.81)	0.9362
Age of menopause q2 vs. q1	1.16 (0.64-2.1)	0.6161	0.76 (0.39-1.48)	0.4188		
Age of menopause q3 vs. q1	0.91 (0.51-1.63)	0.7511	0.63 (0.34-1.17)	0.1429		
Age of menopause q4 vs. q1	0.9 (0.48-1.69)	0.7391	0.67 (0.33-1.37)	0.2762		
Age of menopause missing vs. q1	1.03 (0.59-1.8)	0.9114				
Age of menopause not yet* vs. q1	4.11 (2.41-7.01)	>0.0001				
Age of full-term pregnancy q2 vs. q1	1.55 (1.1-2.2)	0.0133	1.52 (0.82-2.81)	0.1825	1.96 (0.69-5.57)	0.2043
Age of full-term pregnancy q3 vs. q1	1.03 (0.71-1.49)	0.8902	0.94 (0.48-1.85)	0.8659	1.61 (0.62-4.19)	0.327
Age of full-term pregnancy q4 vs. q1	0.91 (0.62-1.33)	0.6154	1.96 (1.01-3.8)	0.0471	0.6 (0.21-1.71)	0.3371
Age of full-term pregnancy missing vs. q1	1.43 (0.95-2.15)	0.0822	1.16 (0.46-2.88)	0.7546	2.64 (0.68-10.25)	0.161
Age of full-term pregnancy not yet** vs. q1	16.5 (5.32-51.21)	>0.0001	N/A	N/A	19.2 (3.57-103.1)	0.0006
Oral contraception usage q2 vs. q1	1 (0.64-1.54)	0.9822	1.29 (0.67-2.48)	0.4473	0.84 (0.31-2.25)	0.7241
Oral contraception usage q3 vs. q1	1.67 (1.11-2.5)	0.0129	1.8 (0.94-3.46)	0.0774	1.45 (0.58-3.63)	0.4289
Oral contraception usage q4 vs. q1	1.34 (0.85-2.1)	0.2049	1.95 (0.92-4.11)	0.0814	0.85 (0.3-2.4)	0.7582
Oral contraception usage missing vs. q1	1.08 (0.71-1.63)	0.7282	0.9 (0.46-1.74)	0.7472	1.44 (0.58-3.59)	0.4294
Mastectomy	0.05 (0.01-0.39)	0.0038	N/A	N/A	N/A	N/A
Oophorectomy	0.18 (0.11-0.29)	>0.0001	0.22 (0.13-0.38)	>0.0001	N/A	N/A

N/A: could not be fit in the model; q1 … q4: first to fourth quartile, with the first being the youngest (~40 years old); *ovulating; **never had full term pregnancy

The ovulating stratum (i.e. “not yet” in the menopausal stage as from Tables 4 and 5) had a higher hazard of breast cancer as compared to the first age quartile of the menopausal stage stratum (i.e. women entering the menopausal stage at ~40 years old). An early age of menopause (first age quartile, ~40 years old) was associated with a higher hazard of breast cancer as compared to an older age of menopause (yet a higher hazard than the ovulating stratum), consistently across all *BRCA1/2* carrier types, in the whole population and in the menopausal stage stratum. Note that menopause may be happening within the same year a chemotherapy was initiated right upon breast cancer diagnosis, resulting *de facto* in competing events (as diagnosis of menopause was given to the nearest year of age). Women who had either oophorectomy had a lower hazard as compared to those who had not (mastectomy could not be properly assessed due to the low number of events).

Finally, when fitting model (vi), i.e. feature-selected Cox regression using a forward/backward stepwise heuristic driven by AIC, for both *BRCA1/2* sets only the year of birth, all the menopausal age stages (along with ovulating stratum), and the oophorectomy variables were selected in the final model (RH were in line with those obtained from other models).

## Discussion

In this study we applied a robust model selection framework composed of linear and non-linear statistical techniques for survival analysis, with the objective to test the predictive ability of existing risk scores for breast cancer in a population of *BRCA1/2* carriers, and to improve over the current state-of-the-art, from the models based on early genotyping and familial assessment to the most recent SNP scoring, trying to combine both clinical/demographic information with high-resolution genetics. Also, we assessed the incidence and the determinants of breast cancer in the study population, and stratified the analyses by the menopausal status.

RSF did not yield higher performance as compared to CPH, even if for some of the data sets the proportional hazard assumption was not met. Interestingly, the re-calibration of GPS *via* the inclusion of SNPs in a CPH did not produce a better model fit (in terms of c-index or AUROC) than using the original GPS in a CPH. In our case, the c-index estimation through out-of-bag distributions may be a conservative choice, but robust to over-training.

This study further highlights the predictive ability of GPS for *BRCA2*, showing an increased RH 1.33 (1.1-1.61) in the whole population, although not significant at the 0.05 level in the menopausal/ovulating stage strata. Instead, for *BRCA1* the effect of GPS was protective (RH = 0.76, p = 0.01) in the whole *BRCA1* population and in the ovulating stage stratum (also protective but not significant at the 0.05 level in the menopausal stratum). Previous findings of Ingham *et al.* [8] already pointed out the predictive ability of 18 SNP GPS in *BRCA2* but not *BRCA1* carriers. This significant association of GPS however was not supported when fitting the stepwise models, retaining only the year of birth, the menopausal stage and the oophorectomy variables (across all carrier types and strata). The age cohort and oophorectomy had been previously associated with increased and decreased risk of breast cancer, respectively [35, 36]. We found that an later ages of menopause have a lower hazard of breast cancer as compared to the first age quartile, ~40 years old, which seems in contradiction with previous results by Tyrer *et al.* [18], and being on the ovulating stratum has a higher hazard than experiencing early menopause. This is likely a model artefact, because the menopause may happen (being induced) right after to the initiation of a chemotherapy (i.e. competing events), and the menopause age is given to the nearest year. In any case, as women entering the menopausal stage early may be subject to treatment for preserving fertility, this warrants further investigation including a number of potential confounders.

Limitations of this study are in the usage of the c-index as a measure of model performance, which presents a series of flaws [37–39], although our results were confirmed using the AUROC estimator. Alternative measures have been presented, like prediction error curves [40] that may be employed as additional indicators. Another limitation is that we did not fit the Cox models using time-updated covariates (as for menopausal stage or age of menarche, for instance) and this may dilute their effect across all time, instead of calculating the hazard on specific time intervals.

## Conclusions

We exploited model selection in machine learning towards the personalised diagnosis of breast cancer, incorporating different domains of information including genetics, clinical, and demographics. Given the improvement in prediction performance obtained by coupling a genetic progression score with clinical and demographic markers, further investigation for identifying both genetic and non-genetic factors (along with their interactions in terms of epigenetics) is warranted.
